# Clinical and Biomechanical Study of Laminoplasty for Thoracic and Lumbar Intradural Tumors

**DOI:** 10.3390/jcm12010355

**Published:** 2023-01-02

**Authors:** Lijun Jiang, Jie Luo, Haiyi Gong, Fei Zhang, Linxiang Zhang, Linfei Cheng, Xin Gao, Dan Zhang, Tielong Liu, Jianru Xiao

**Affiliations:** 1 School of Medicine, Ningbo University, Ningbo 315211, China; 2 Orthopaedic Oncology Center, Department of Orthopedics, Changzheng Hospital, Naval Medical University, Shanghai 200003, China; 3 Department of Orthopedics, Ningbo Beilun Orthopedic Hospital, Ningbo 315899, China

**Keywords:** intraspinal tumors, intradural tumors, laminoplasty, laminectomy, range of motion (ROM), cerebrospinal fluid (CSF), stability, bony fusion, spinous processand lamina complex

## Abstract

(1) Background: Primary intraspinal tumors account for 2–15% of all central nervous system (CNS) tumors. Most intraspinal tumors are benign, and about 40% of them occur intradurally, for which early surgery is the preferred treatment. Laminectomy with pedicle screw fixation is the conventional surgical treatment. However, laminectomy with pedicle screw fixation is likely to reduce the spinal range of motion (ROM), with many other complications, although it can maintain the stability of the spine. The aim of this study is to determine whether laminoplasty as a new surgical approach for thoracic and lumbar intradural tumors is superior to laminectomy in preserving spinal ROM, maintaining spinal stability and reducing postoperative complications. (2) Methods: We retrospectively analyzed 50 patients who received intradural tumor resection, including 23 who received traditional laminectomy with pedicle screw fixation and 27 who received new laminoplasty. Spinal ROM was evaluated by lumbar flexion/extension radiograph and biomechanical evaluation. Spinal stability was evaluated by imaging observations of the spinal Cobb angle and laminar bone fusion. Postoperative complications were evaluated according to cerebrospinal fluid (CSF) leakage and the length of hospital stay. (3) Results: Compared with the laminectomy group, patients in the laminoplasty group exhibited a better spinal ROM (31.6 ± 12.0° vs. 21.7 ± 11.8°, *p* = 0.013), a smaller Cobb angle (9.6 ± 4.3 vs. 12.5 ± 5.3, *p* = 0.034), a lower incidence of CSF leakage (4/14.8% vs. 11/47.8%, *p* = 0.015), and a shorter length of hospital stay (13.1 ± 1.8 vs. 15.1 ± 2.3 days, *p* = 0.001). Most patients in the laminoplasty group had satisfactory bone fusion. The biomechanical experiment also demonstrated that spinal ROM in laminoplasty was larger than that in the laminectomy group. (4) Conclusions: Compared with the traditional surgery, the new laminoplasty surgery can better maintain the stability of the spine, preserve spinal ROM, and reduce postoperative complications. It is a surgical method that can be clinically popularized.

## 1. Introduction

Primary intraspinal tumors refer to tumors in the spinal canal from the level of the foramen magnum to the coccyx, accounting for 2–15% of all central nervous system (CNS) tumors [1,2]. Primary intraspinal tumors usually manifest with pain and neurological deficits [1], and most are benign tumors with well-defined boundaries [3]. About 40% of intraspinal tumors occur intradurally [2], for which early surgery is the preferred treatment and radical resection can usually be curative [2,3,4,5,6].

There are two main surgical methods for intradural spinal tumor resection. One is laminectomy with pedicle screw fixation [7,8], in which the insertion of pedicle screws can enhance the stability of the spine and prevent spinal deformity [6,7]. However, laminectomy with pedicle screw fixation causes a significant decrease in the range of motion (ROM) of the spine [9,10], and the resection of spinal posterior natural structure may result in postoperative scar tissue growth in the spinal canal, scar adhesion, iatrogenic spinal stenosis, and other complications [8]. The other surgical method is laminoplasty. Ever since laminoplasty was introduced by Raimondi et al. in 1976 [11], various options for laminoplasty have been reported. Laminoplasty can maximally preserve the natural structure of the posterior spine, reduce surgical trauma and scar tissue formation in the spinal canal, preserve spinal ROM, and maintain spinal stability [7,12,13,14,15]. A meta-analysis involving 1096 patients suggested that laminoplasty might be a safer and more effective surgical method in the treatment of spinal cord tumors [16]. Although laminoplasty is employed mostly in cervical spinal surgery, it can also be applied to thoracic and lumbar surgery [15].

In recent years, laminoplasty has more commonly been used for the clinical treatment of intradural tumors as compared with laminectomy with pedicle screw fixation. The differences between the two surgical modalities have been discussed in some studies [6,8,16,17,18]. Laminectomy with pedicle screw fixation has certain disadvantages and complications, but few studies in the literature have fully addressed thoracic and lumbar laminoplasty versus laminectomy with pedicle screw fixation. Notably, there is a lack of studies on postoperative spinal ROM, spinal stability, and complications. The aim of this study is to determine whether laminoplasty, as a new surgical approach for thoracic and lumbar intradural tumors, is superior to laminectomy in preserving spinal ROM, maintaining spinal stability, and reducing postoperative complications

## 2. Materials and Methods

**Figure 1:**
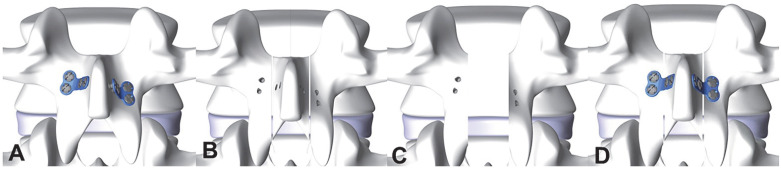
Procedures for resection and replantation of the spinous process and lamina complex. (**A**) The microplates were prefixed on the lamina and spinous process on both sides of the target plane; (**B**) the prefixed microplates and screws were removed, and laminectomy was performed using an ultrasonic osteotome; (**C**) the spinous process and lamina complex were removed and the tumor was resected; and (**D**) the spinous process and lamina complex were reduced and fixed with microplates. Finally, the supraspinous ligament at the head and tail of the lamina was sutured.

We retrospectively analyzed 50 patients with thoracic and lumbar intradural tumors who received tumor resection in the Department of Orthopedic Oncology at Changzheng Hospital of the Naval Medical University (Shanghai, China) between April 2020 and September 2021. Of them, 27 patients who received intradural spinal tumor resection by new laminoplasty with microplate fixation were designated as the laminoplasty group (Figure 1), and the remaining 23 patients who received intradural spinal tumor resection by traditional laminectomy with pedicle screw fixation were designated as the laminectomy group. This study was approved by the ethics committee of the hospital, and informed consent was obtained from each patient before the initiation of the study.

The inclusion criteria were: (1) patients with intradural tumors who received operation for the first time; (2) patients with no spinal structure destruction or instability; (3) patients with complete clinicopathological data; and (4) patients who were followed up for at least one year. The exclusion criteria were: (1) patients whose facet joint was destroyed; (2) patients with recurrent tumors; and (3) patients whose lesions had caused spinal destruction and instability. All patients were followed up for at least one year.

Cases in which the fixed segment included the lumbar spine were screened, and lumbar flexion/extension radiograph was performed at 6 months after operation to measure spinal ROM. Two straight lines made through and parallel to the upper edge of the L1 and S1 vertebral bodies, respectively; two vertical lines made on the above two lines were intersected; and the angle that was formed was measured by using a protractor. The difference between the angle measured in flexion and the one measured in extension corresponded to the lumbar ROM.

At 1 week, 3 months, 6 months, and 12 months after operation, frontal and lateral X-ray radiography of the spine was performed to check the sagittal alignment of the spine, and the postoperative stability of the spine was evaluated by measuring the Cobb angle of the fixed segment. For cases with only one fixed segment, the Cobb angle was measured between the upper endplate of the last vertebral body and the lower endplate of the next vertebral body at the fixed level. A CT scan was performed at 6 and 12 months after surgery to evaluate the laminar bone fusion.

A comprehensive approach was adopted to diagnose the occurrence of postoperative cerebrospinal fluid (CSF) leakage. The diagnosis of postoperative CSF leakage should meet one of the following criteria: (1) the mean volume of drainage within 3 postoperative days is greater than 150 mL; (2) the presence of postural headache after surgery; (3) the wound’s postoperatively continuing to drain clear fluid; or (4) the formation of a pseudomeningocele or CSF leakage through the incision.

The surgical time, intraoperative blood loss, and length of hospital stay were summarized, calculated, and compared. Before operation and at 3 and 12 months after operation, lower-back pain and lower-limb pain were assessed by visual analog scale (VAS), and the neurological function was evaluated by the Oswestry disability index (ODI). An MRI was performed 6 months after surgery to detect tumor recurrence and scar adhesions in the spinal canal and the ligaments’ repair.

**Figure 2:**
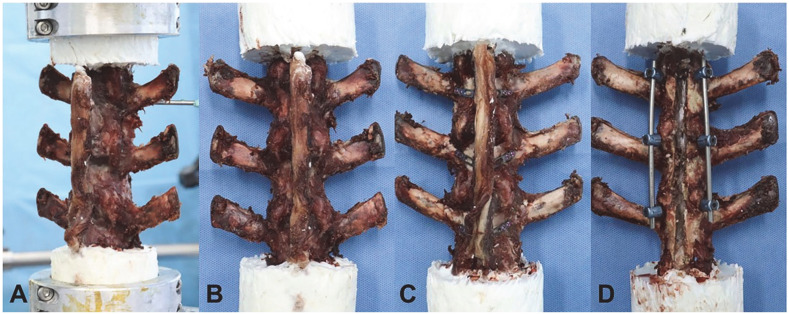
The mechanical testing machine and specimens for biomechanical experiments. (**A**) Normal lumbar spine specimens were fixed on a 6-degrees-of-freedom mechanical testing machine; (**B**) control group—normal specimens; (**C**) laminoplasty group—L2, L3 and L4 laminoplasty with microplate fixation; and (**D**) laminectomy group—L2, L3 and L4 laminectomy with pedicle screw fixation.

In addition to the lumbar flexion/extension radiograph, this biomechanical test is also a way to determine the spinal ROM. In this study, 18 fresh-frozen goat lumbar spine specimens of similar size were used. All specimens were examined by anteroposterior and lateral radiographs to exclude fractures, deformities, and other abnormal spinal lesions. Prior to testing, the specimens were thawed and stripped of muscle tissue, while the spinal ligaments, joint capsules, intervertebral discs, and bony structures were carefully retained. Segments L1–L5 of the specimen were intercepted on a 6-degrees-of-freedom mechanical testing machine equipped with an optical tracking system with motion capture markers, a motion capture camera, and optical tracking software. The upper and lower ends of the specimen were fixed on a mechanical testing machine, with motion capture markers rigidly fixed at the level of the L2 vertebral body. A computer data analysis and processing system was used to evaluate the ROM of specimens L2–L4. The specimens were divided into three groups: a control group of normal specimens; a laminectomy group of L2, L3, and L4 laminectomy with pedicle screw fixation; and a laminoplasty group of L2, L3, and L4 laminoplasty with microplate fixation (Figure 2). All specimens were tested under three loading conditions, including flexion–extension (FE), lateral bending (LB), and axial rotation (AR). In each direction, the mechanical testing machine applied three 7.5 Nm loading cycles to the specimen, and the data of the third loading cycle were recorded. The ROM of L2–L4 of each specimen in each direction was obtained by software analysis and data processing.

### Statistical Analysis

Statistical analysis was performed using IBM SPSS (SPSS Statistics V26; SPSS, Inc., an IBM Company, Chicago, IL, USA). Continuous variables are presented as the mean ± standard deviation (SD) and were compared by using a Student’s *t* test. The three sets of continuous variables were compared by ANOVA (analysis of variance). Categorical variables were compared by using a χ^2^ test. A *p*-value of less than 0.05 was considered statistically significant.

## 3. Results

There was no statistically significant difference in the baseline characteristics between the two groups (Table 1). Compared with the laminectomy group, the laminoplasty group had many advantages (Table 2).

### 3.1. Larger Spinal ROM and Better Stability in Laminoplasty Group

**Figure 3:**
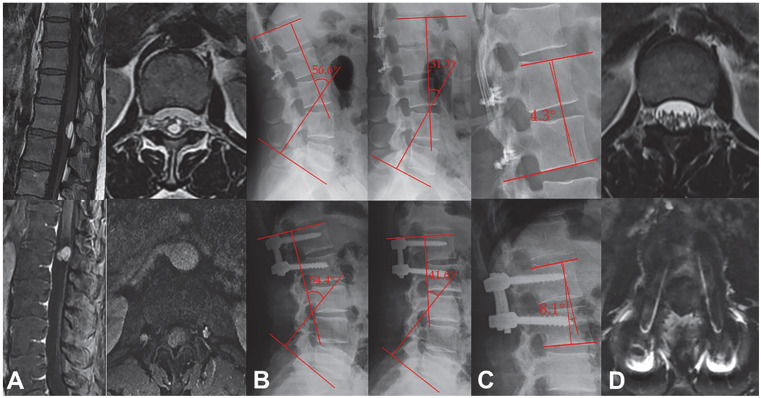
Radiographic findings were compared between a 38-year-old female patient in laminoplasty group and a 50-year-old female patient in laminectomy group. (**A**) Preoperative MRI identified the tumor at L1 level in the patient in laminoplasty group and the tumor at L1–L2 level in the patient in laminectomy group. (**B**) Postoperative L-spine lordotic kyphotic X-ray film showed that the lumbar ROM of the patient in laminoplasty group was 25.3° vs. 12.8° in laminectomy group. (**C**) Postoperative frontal and lateral X-ray film showed that the Cobb angle of the fixed segment was 4.3° in the patient in the laminoplasty group vs. 8.1° in the patient in laminectomy group. (**D**) Postoperative MRI identified no tumor recurrence in either group; there were no intraspinal scar adhesions or restenosis in the patient in laminoplasty group; imaging of the patient in laminectomy group showed that the vertebral canal shape was irregular; and the dural sac was partially compressed.

**Figure 4:**
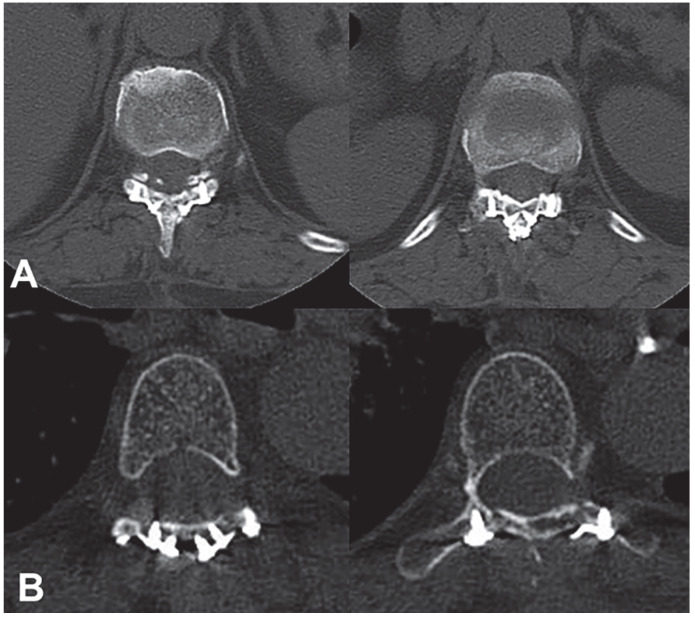
Bone fusion of the replanted lamina. (**A**) In a 55-year-old female patient, postoperative CT scan showed favorable bony fusion of the replanted lamina; (**B**) another 56-year-old female patient also had favorable bone fusion.

Compared with the laminectomy group, patients in the laminoplasty group showed a larger spinal ROM at 6 months after surgery (31.6 ± 12.0° vs. 21.7 ± 11.8°, *p* = 0.013) (Table 3; Figure 3) and a smaller Cobb angle (9.6 ± 4.3 vs. 12.5 ± 5.3; *p* = 0.034) at 12 months after surgery (Figure 3). CT scans showed that most patients achieved complete laminar bone fusion, and in no patient was it found that the bilateral laminar incision line did not achieve bone fusion, but five patients achieved partial laminar bone fusion (in a certain segment, the unilateral laminar incision line did not achieve bone fusion) (Figure 4).

### 3.2. Better Pain Releif and Improved Neurological Function in Laminoplasty Group

The VAS and ODI of both groups decreased over time, showing significant improvements over those from before the operation. At 12 months after the operation, there were significant differences in VAS and ODI between the two groups, showing that patients in the laminoplasty group had lower VAS (1.0 ± 0.6 vs. 1.3 ± 0.6, *p* = 0.032) and lower ODI (10.2 ± 3.5 vs. 12.4 ± 3.3; *p* = 0.026).

### 3.3. Less Trauma and Faster Postoperative Recovery in Laminoplasty Group

Compared with the laminectomy group, patients in the laminoplasty group had lower intraoperative blood loss (275.6 ± 146.3 mL vs. 363.5 ± 143.2 mL, *p* = 0.038), a lower mean volume of drainage (94.9 ± 44.0 mL vs. 154.2 ± 87.3 mL, *p* = 0.003), and a shorter length of hospital stay (13.1 ± 1.8 vs. 15.1 ± 2.3 days, *p* = 0.001). There was no significant statistical difference in surgical time between the two groups (207.2 ± 62.9 vs. 191.7 ± 59.3, *p* = 0.378).

### 3.4. Reduced Incidence of CSF Leakage in Laminoplasty Group

As mentioned above, patients in the laminoplasty group had a lower mean volume of drainage. The mean volume of drainage was more than 150 mL in four patients (14.8%) in the laminoplasty group vs. 11 patients (47.8%) in the laminectomy group (*p* = 0.015). The color of the drainage was clear in four patients (14.8%) in the laminoplasty group vs. 10 patients (43.5%) in the laminectomy group (*p* = 0.031). Postural headache occurred in two patients (7.4%) in the laminoplasty group vs. eight patients (34.8%) in the laminectomy group (*p* = 0.030). No pseudomeningocele or incisional cerebrospinal fluid leakage occurred. According to our criteria, the incidence of CSF leakage was lower in the laminoplasty group, with four patients (14.8%) having CSF leakage vs. 11 patients (47.8%) in the laminectomy group (0.015).

### 3.5. Results of the Biomechanical Experiments

As shown in Table 4, there were significant differences in FE motion between the control, laminoplasty, and laminectomy groups (F = 362.5, *p* < 0.05). Compared with the control group, spinal ROM decreased by 10.20% in the laminoplasty group and 79.67% in the laminectomy group (*p* < 0.05). There were also significant differences in LB motion between the three groups (F = 616.2, *p* < 0.05). Compared with the control group, ROM decreased by 24.10% in the laminoplasty group and 81.92% in the laminectomy group (*p* < 0.05). In addition, there were significant differences in AR motion between the three groups (F = 143.3, *p* < 0.05). Compared with the control group, ROM decreased by 15.77% in the laminoplasty group and 55.88% in the laminectomy group (*p* < 0.05) (Table 4).

MRI indicated no tumor recurrence and no intraspinal restenosis or scar adhesions in the laminoplasty group. There was also no tumor recurrence in the laminectomy group, but a few patients had scar adhesion in the spinal canal.

## 4. Discussion

The incidence of intraspinal tumors ranges from 0.3 to 1.6 per 100,000 people per year and seems to increase over time. About 40% intraspinal tumors occur intradurally [2]. Most intradural tumors are benign, and pain is the most common symptom [4]. Complete surgical resection is the most effective treatment for intradural tumors. Laminectomy with pedicle screw fixation and laminoplasty are the main two surgical methods. Laminectomy is the mainstay of surgical treatment at present, but it has certain drawbacks owing to the removal of the posterior natural structure of the spine and the use of screws for fixation. Laminoplasty has been widely used in recent years. Various solutions have been proposed to replant the lamina. Liu et al. [19] reported that the lamina could be fixed by sutures. However, the fixation strength of the sutures could not be guaranteed, and the increased time needed for external fixation and bed rest after surgery would preclude early mobilization and rehabilitation. In addition, sutures are not appropriate for multilevel laminoplasty. Park et al. [20] reported that lamina screws could be used to fix the lamina, but the thickness of the lamina screws needs to match the thickness of the lamina, and they cannot be directly and universally used to fix the spinous process and lamina complex, as this may lead to displacement and nonunion after fixation of the spinous process and lamina complex [14]. Combining the fixation system of laminar screws with pedicle screws is somewhat complex in operation, and the screw-rod system may not fit the posterior muscles and soft tissues, leading to lower-back discomfort for patients after operation. Although the use of laminoplasty combined with microplate fixation has been reported in the cervical spine in multiple studies [6,7,8,14,15], few studies have reported its use in the thoracolumbar spine. Because the microplate can directly fix the spinous process and lamina complex and can simply and plastically fit the posterior muscles and soft tissues, it has been widely used in laminoplasty. Biomechanical testing has demonstrated that laminoplasty combined with microplate fixation can improve the stability, compressive resistance, and resistance to bending, shear, and the rotation of the spine [7]. The microplate fixation provides favorable conditions for good prognoses for patients with laminoplasty.

Total laminectomy may cause the loss of the bony structures and ligaments at the posterior aspect of the spine. Scar tissue growth in the spinal canal after laminectomy may lead to iatrogenic spinal stenosis [15]. It can also affect the exposure of secondary surgery, thus increasing the risk of secondary surgery [21]. Efforts have been made to avoid epidural adhesion and compression due to postoperative scar tissue growth, including the intraoperative instillation of anti-inflammatory drugs and steroid drugs and the use of silicon membranes, free adipose tissue, preadipocytes, and silicone rubber sheets as a barrier behind the dura, but with poor outcomes [22,23,24,25]. At the same time, rejection reaction and infection are also problems caused by the implantation of a foreign barrier. In our study, no intraspinal scar adhesions or restenosis were observed in the laminoplasty group, indicating that given that the spinous process and lamina complex is a natural component of the spine, returning it to its original place as the posterior dura barrier would be the optimal choice [15].

In 1984, Denis et al. [26] proposed the classic three-column theory of the spine, knowing that the posterior column plays an important role in maintaining spinal stability [27]. Mullin et al. [28] followed up with the patients who received lumbar laminectomy for a mean of 31 months after surgery and found that 54% patients showed radiographic signs of instability, after the authors excluded patients who had undergone fusion and other unknown conditions. Papagelopoulo et al. [29] reported their 14-year follow-up results after multilevel lumbar or thoracolumbar total laminectomy in patients with benign intraspinal tumors, showing that spinal deformities occurred in 33% of children and adolescents and 8% of young adults, with an increased incidence in patients who had more than two laminae removed. Spinal instability can lead to a series of problems, such as lower-back pain and kyphosis, seriously affecting the prognosis of patients [30,31]. Laminoplasty can reconstruct the natural structure of the posterior spine, and pedicle screws provide direct rigid mechanical fixation of the spine. Both methods can maintain the stability of the spine. Zhang et al. [12] reported that pedicle screws could achieve three-column fixation because of their high biomechanical strength, while small plates combined with monocortical screws have weak fixation strength for the spinous process and lamina complex. However, our study demonstrated that the postoperative Cobb angle of the spine in the laminoplasty group was smaller than that in the laminectomy group, which was closely related to the reconstruction of the posterior column and the preservation of the attachment point of the sacrospinal muscle. The integrity of the natural structure of the spine is more conducive to the maintenance of spinal stability.

Pedicle screws have biomechanical strength and can to a certain extent maintain spinal stability. However, at the same time, they significantly limit the postoperative ROM of the lumbar spine [9,10]. Long segmental pedicle screw fixation can cause segmental spinal movement disorder [7]. At the same time, the limited ROM of the fixed segment will aggravate the acceleration of spinal degeneration in the adjacent segment. At present, there is a lack of statistical research on the effect of laminectomy combined with the microplate fixation on spinal mobility. However, the limitation of postoperative spinal mobility will affect the short-term quality of rehabilitation training and affect the prognosis and quality of life of the patients in the long term. In this study, we postoperatively performed imaging and biomechanical testing to evaluate the postoperative spinal ROM in patients in the two groups. Compared with the laminectomy group, patients in the laminoplasty group had a significantly larger postoperative spinal ROM. The biomechanical experiment also showed that the ROM of the spine was decreased in the two surgical groups as compared with that in the normal control, and the decrease was more pronounced in the laminectomy group, indicating that both surgical methods can provide fusion and fixation of the spine but provide no increase in the ROM of the spine and abnormal spinal movement. On this premise, the spinal ROM was preserved to the greatest extent in the laminoplasty group; therefore, we believe that this surgical modality is more beneficial to improving the short-term recovery and long-term prognosis of patients.

Bone fusion of the lamina after laminoplasty is an important observation index. There is a close relationship between the tool of laminectomy and bone fusion of the lamina. We used an ultrasonic osteotome to incise the lamina, knowing that the ultrathin cutting head of the ultrasonic osteotome can reduce the bone loss caused by laminectomy and effectively promote bone healing in the lamina. At the same time, it can significantly shorten the surgical time and reduce intraoperative blood loss and the risk of injury to the surrounding soft tissues and key structures, such as the nerves and blood vessels [32,33]. Previous studies have demonstrated that satisfactory bone fusion of the laminae can be achieved in about 6 months or 1 year after surgery. Our follow-up study showed that although favorable bone fusion was achieved in most patients, five patients achieved only partial laminar bone fusion, probably due to the following three factors: (1) There was still partial bone loss during laminectomy. After reset and fixation, the bone surface on both sides of the incision line did not fully fit, thus affecting the healing. (2) As the spinous process and lamina complex were completely removed and the attached muscles, ligaments, and other tissues were all stripped off, the blood supply of the spinous process and lamina complex was destroyed. (3) The follow-up time was limited. Allograft bone implantation and the replantation of the pedicled spinous process and lamina complex may be able to promote bone fusion, but only one end of the pedicled spinous process and laminar complex was dissected without dissecting the other end, and the tumor was exposed by rotating the complex.

CSF leakage is a recognized complication after intradural surgery [34], which may induce a series of adverse events during postoperative recovery, including postural headache, pseudomeningocele or fistula formation, poor wound healing, infection, meningitis, arachnoiditis, and even spinal cord abscess [34,35]. An overview of the literature reveals that the incidence of CSF leakage after laminectomy and laminoplasty is relatively low [7,8,12,19]. As the diagnostic criteria of CSF leakage may affect the true incidence, the detection of postoperative CSF leakage requires further comprehensive evaluation [36]. The generally accepted diagnostic criterion of CSF leakage is the presence of CSF leakage from the incision [34]. However, a postoperative ambulation of the patient, the full replenishment of CSF, and the physiological pressure of CSF on the dural suture may weaken the suture or even cause partial CSF leakage [37]. At present, with a tight fascial suture and drainage tube placement, the risk of incisional CSF leakage is relatively low, but clear drainage fluid may flow out of the drainage tube, resulting in increased postoperative drainage volume and postural headache with low intracranial pressure. Postural headache is considered important evidence for the diagnosis of CSF leakage [38]. To screen all cases that may be at risk of CSF leakage, we set four observation indicators for CSF leakage in this study. CSF leakage was defined when any of the four indicators had been met. It was found in our study that both the incidence of CSF leakage and the amount of drainage in the laminoplasty group were significantly lower compared to those in the laminectomy group and that the incidence of orthostatic headache was also significantly reduced, thus shortening the confinement to bed and accelerating the postoperative recovery of the patients. According to our analysis, the reduction in the incidence of CSF leakage may be related to the coverage of the spinous process and lamina complex at the sutures of the dorsal dura incision and the reattachment of the paraspinous muscle to the replanted spinous process.

## 5. Conclusions

Laminoplasty has been increasingly used for the surgical resection of intradural tumors in the thoracic and lumbar spine as compared with conventional laminectomy with pedicle screw fixation, as this new surgical modality can better maintain the stability of the spine, preserve spinal ROM, and reduce postoperative complications. In addition, it can ensure good short-term recovery for patients. It is a surgical method that can be clinically popularized.

## Figures and Tables

**Table 1:** Baseline characteristics of the enrolled patients.

	Laminoplasty (*n* = 27)	Laminectomy (*n* = 23)	*p*
Age (y)	47 ± 15	51 ± 14	0.254
Gender (female)	12(44%)	15(65%)	0.166
Pathological diagnosis (schwannoma/meningioma/others)	11/6/10	13/5/5	0.446
Fixation segments (1/2/3/>3)	2/13/8/4	0/7/14/2	0.121
Fixation position (thoracic/thoracolumbar/lumbar)	7/9/11	4/6/13	0.529

**Table 2:** Comparison of the surgical and follow-up results between the two groups.

Results	Laminoplasty (*n* = 27)	Laminectomy (*n* = 23)	*p*
Surgical time (min)	207.2 ± 62.9	191.7 ± 59.3	0.378
Intraoperative blood loss (mL)	275.6 ± 146.3	363.5 ± 143.2	0.038
Mean volume of drainage (mL)	94.9 ± 44.0	154.2 ± 87.3	0.003
Color of drainage (clear)	4(14.8%)	10(43.5%)	0.031
Postural headache	2(7.4%)	8(34.8%)	0.030
Hospital stay (days)	13.1 ± 1.8	15.1 ± 2.3	0.001
CSF leak	4(14.8%)	11(47.8%)	0.015
VAS			
Before operation	6.9 ± 2.3	6.5 ± 2.0	0.516
3 months after surgery	2.4 ± 0.8	2.2 ± 0.8	0.419
12 months after surgery	1.0 ± 0.6	1.3 ± 0.6	0.032
ODI (%)			
Before operation	68.9 ± 13.4	66.1 ± 14.3	0.467
3 months after surgery	25.2 ± 6.6	24.9 ± 6.1	0.862
12 months after surgery	10.2 ± 3.5	12.4 ± 3.3	0.026
Cobb angle (°)			
Before operation	9.1 ± 4.8	9.7 ± 5.9	0.668
3 months after surgery	8.1 ± 3.0	11.2 ± 6.8	0.040
12 months after surgery	9.6 ± 4.3	12.5 ± 5.3	0.034

ODI, Oswestry disability index; VAS, visual analog scale; CSF, cerebrospinal fluid.

**Table 3:** Comparison of spinal ROM between the two groups.

	Laminoplasty (*n* = 20)	Laminectomy (*n* = 19)	*p*
ROM (°)	31.6 ± 12.0	21.7 ± 11.8	0.013

ROM, range of motion.

**Table 4:** Comparison of spinal ROM between laminoplasty and laminectomy groups.

	Intact	Laminoplasty	Laminectomy
FE	14.41 ± 1.19	12.94 ± 0.67	2.93 ± 0.24
LB	17.26 ± 1.09	13.10 ± 0.54	3.12 ± 0.24
AR	13.19 ± 1.22	11.11 ± 0.41	5.82 ± 0.38

FE, flexion–extension; LB, lateral bending; AR, axial rotation.

## Data Availability

The data presented in this study are available on request from the corresponding author. The data are not publicly available, owing to privacy restrictions.
